# Design of Cost-Effective QC Procedures for Clinical Chemistry Assays

**DOI:** 10.6028/jres.093.022

**Published:** 1988-06-01

**Authors:** James O. Westgard

**Affiliations:** Department of Pathology and Laboratory Medicine, Medical School, Medical Technology Program, School of Allied Health Professions, University of Wisconsin, Madison, WI

## Introduction

In clinical laboratories, the major production processes are analytical processes. Quality management strategies must include approaches for optimizing the cost-effectiveness of analytical processes. “Cost-effective QC,” in this context, is concerned with selecting or designing a quality control procedure that maximizes both the quality and productivity of an analytical process [1]. There are many factors that need to be considered, including the medically required quality for the analyte being measured, the characteristics of the measurement procedure (type of process or system, precision, accuracy, drift stability, and frequency of occurrence of errors), the type and structure of the errors occurring (random or systematic, intermittent or persistent), and the error detection and false rejection characteristics of the control procedure itself (probabilities for rejection, average run lengths).

To assess the possible interactions of these many factors, it is necessary to have a “design tool” such as a quality-productivity model, which is based on the industrial concept of quality-costs. With the aid of such models, the effects of these many factors can be predicted, facilitating the planning of analytical processes that will provide cost-effective operation.

## Quality-Productivity Models

The industrial concept of “quality costs” was described by Feigenbaum in 1956 [2]. Quality costs include components of prevention costs, appraisal costs, and failure costs, including both internal and external failure costs. When costs are restricted to process costs in terms of samples analyzed or repeated, prevention costs are losses of samples for calibration, and appraisal costs are losses for analyzing control samples, both of which can be estimated from knowledge of the physical variables of the analytical system and process. Failure costs are losses for repeat runs and repeat requests, which can be predicted from performance characteristics of the measurement and control procedures. These losses, described in terms of the process variables and characteristics, provide an estimate of process waste. Test yield, or productivity, is estimated by the difference between the maximum output and the process waste (1 − [average quality costs or losses]).

Table 1 provides an example quality-productivity model for a batch analytical process subject to intermittent errors.

## Example Application

Let us assume a batch process having 30 samples per batch that is calibrated once a week, requiring six calibrators (*C*) be analyzed in duplicate. Assume a workload requiring one run per day for 6 days in a week, thus (*C*) averages one per run for this example. We then postulate a design for a control procedure, enter the number of control measurements per run (*N*), and the probabilities for false rejection and error detection (for medically important errors), as determined from power function graphs obtained by a computer simulation program [3]. Complete details of this example are found elsewhere [1].

**Figure 1:**
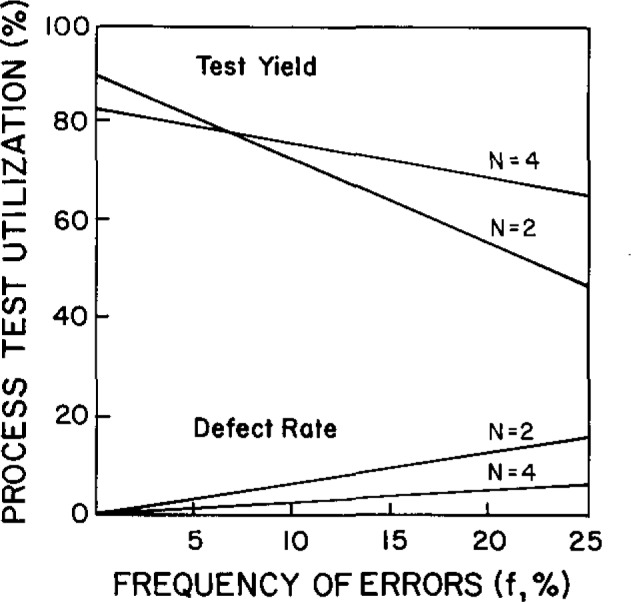
Comparison of quality (defect rate) and productivity (test yield) for a batch analytical process using a multi-rule control procedure with *N* = 2 and *N* = 4. From reference [1].

Figure 1 shows the performance of a multi-rule procedure [4] for *N* = 2 and *N* = 4. For an unstable measurement procedure having a frequency of errors of 0.10 (or 10%), doing more quality control actually provides higher quality and higher productivity. Doubling the number of controls per run actually reduces the cost. For a stable measurement procedure, with a frequency of errors of 0.01 (or 1%), higher productivity is achieved with *N* = 2.

**Figure 2:**
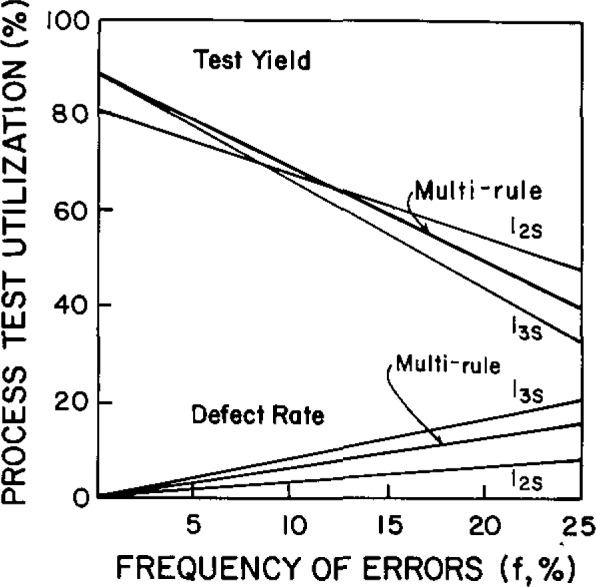
Comparison of quality (defect rate) and productivity (test yield) for a batch analytical process with different control rules all using *N* = 2 (2 control measurements per run). From reference [1].

Figure 2 compares the performance of three different control procedures having *N*=2. For a stable measurement procedure, the use of 3s control limits (1_3s_ control rule) or a multi-rule control procedure [3] provides high quality (low defect rate) and high productivity (high test yield). For an unstable measurement procedure, say a frequency of errors of 0.20 (or 20%), the use of 2s control limits (1_2s_ control rule) provides better quality and higher productivity.

**Figure 3:**
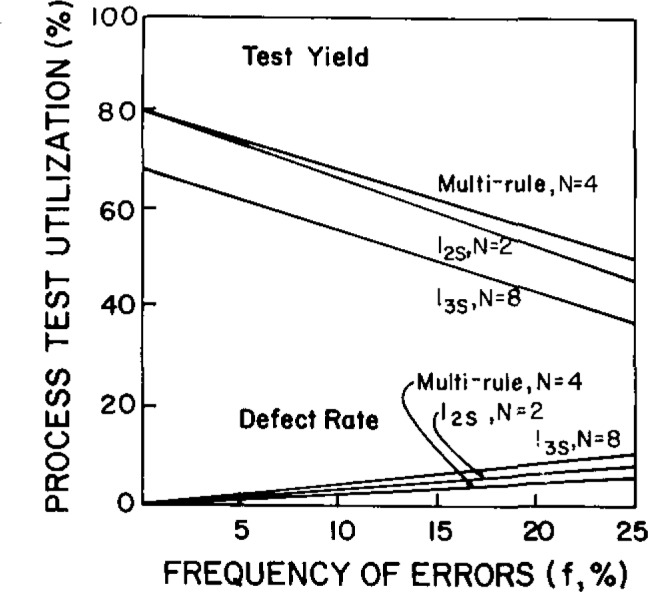
Comparison of quality (defect rate) and productivity (test yield) for a batch analytical process with different control rules and different *N*’s. From reference [1].

Figure 3 shows a comparison of three different control procedures, with different rules and with different *N’*s, all giving nearly the same quality or defect rates. Multi-rule with *N*=4 or 1_2s_ with *N* = 2 provide more cost-effective operation than 1_3s_ with *N* = 8.

## Implications for Selecting and Designing QC Procedures

Given the practical limitations of two to four control measurements per run due to short runs for fast turnaround of results, it is impossible to have an ideal control procedure with *both* high error detection and low false rejection. However, a laboratory can have *either* high error detection or low false rejection if it knows which is more important for the particular application.

Because the performance of an analytical process depends on its stability, i.e., its frequency of errors (*f*), the control procedure can be tailored to fit that frequency of errors. For measurement procedures having a low frequency of errors, the QC procedure should be selected or designed primarily for a low probability of false rejection, with secondary concern for error detection (“low-*f* design”). When there is a high frequency of errors, the QC procedure should be selected or designed for high error detection, with little regard for its probability for false rejection (“high-*f* design”).

One useful strategy is to change control procedures depending on the recent history of the measurement procedure, switching from “low-*f*” to “high-*f*” designs, as necessary. Whenever the analyst is suspicious that performance is deteriorating, the “high-*f*” design can be used. When the process is running smoothly, the “low-*f*” design can be used.

Another strategy is to systematically switch from one design to another as the analytical process goes through its normal operational cycle. For example, at the beginning of the day, test the process carefully using a “start-up” design having high error detection; following successful completion of that testing, switch to a “monitoring” design having very low false rejection; and, over the course of the day, use a “prospective” design to analyze the accumulated control measurements and trigger preventive maintenance procedures prior to tomorrow’s run. Such “multi-stage” designs provide a systematic tailoring of the control procedure to fit the changing operation conditions of the measurement procedure.

Doing “cost-effective QC” in clinical laboratories means using individualized designs of QC procedures that fit the measurement procedures to be monitored. A comprehensive design tool would be useful to help users study and plan their analytical processes. It should provide a wide variety of control rules, incorporate computer simulation to provide the performance characteristics of the selected QC procedure, utilize more realistic error distributions to represent stable and unstable performance, offer choices of intermittent and/or persistent error conditions, and permit input of information on the factors and variables needed to customize the quality-productivity model to fit individual analytical systems.

Routine implementation of such individualized QC designs also would be aided by more advanced QC programs in laboratory information systems. The programs should permit analysts to choose the control rules and the number of control measurements, to select how the rules are applied across materials and/or runs, to specify whether the control signal is a rejection or warning for preventive maintenance, and to define two or more QC procedures that could be employed on a single measurement procedure.

## Figures and Tables

**Table 1:** A quality-productivity model for describing the quality and productivity of a batch analytical process subject to intermittent analytical errors

Measure of quality is “defect rate” $\text{Defect Rate}=f\left(1-P_{\text{ed}}\right)$
Measure of productivity is “test yield”
$\begin{array}{l} \text{Test Yield}=1-\frac{C+N}{T}-\frac{Sp}{T}[R_{\text{tr}}fP_{\text{ed}}+R_{\text{fr}}(1-f)P_{\text{fr}}\\ \;+R_{\text{fa}}f(1-P_{\text{ed}})+R_{\text{ta}}f(1-P_{\text{ed}})(1-f)(1-P_{\text{fr}})] \end{array}$
where the terms are characteristics of the measurement and control procedures, and laboratory policies for repeat runs
*f* is the frequency of occurrence of errors
*P*_ed_ is the probability for error detection
*P*_fr_ is the probability for false rejection
*C* is the number of calibrators
*N* is the number of control measurements per run
*Sp* is the number of patient specimens
*T* is the total samples in a run (*C*+*N*+*Sp*)
*R* terms are rerun factors for true reject, false reject, false accept, and true accept runs
